# LONGITUDINAL USE TRAJECTORIES OF SEDATIVE-HYPNOTICS AND RISK FOR DEMENTIA AMONG MEDICARE BENEFICIARIES

**DOI:** 10.1093/geroni/igad104.1283

**Published:** 2023-12-21

**Authors:** Christopher Kaufmann, Bobby Jones, Grace Hsin-Min Wang, Deanna Fernandes, Ronald Shorr, Wei-Hsuan Lo-Ciganic

**Affiliations:** University of Florida College of Medicine, Gainesville, Florida, United States; University of Florida College of Pharmacy, Gainesville, Florida, United States; University of Florida College of Pharmacy, Gainesville, Florida, United States; University of Florida, Gainesville, Florida, United States; Geriatric Research, Education and Clinical Center, North Florida/South Georgia Veterans Health System, Gainesville, Florida, United States; University of Florida College of Pharmacy, Gainesville, Florida, United States

## Abstract

Research suggests use of sedative-hypnotic medications (Benzodiazepines [BZDs; e.g., diazepam] and non-BZD hypnotics [e.g., zolpidem]) to be associated with incident Alzheimer’s disease and related dementias (ADRD). We identified longitudinal dose and duration use patterns of these medications and their associations with ADRD risk. This retrospective cohort study used 2016-2018 claims data from a 15% national sample of fee-for-service Medicare beneficiaries and included beneficiaries aged ≥65 years initiating use of a) BZDs only, b) non-BZDs only, or c) BZDs+non-BZDs concurrently. We excluded beneficiaries with ADRD diagnosis 6 months before and 12 months after first BZD/non-BZD prescription date. Based on average weekly diazepam milligram equivalent dose (low: <15, moderate: 15-30, high: >30), we used group-based multi-trajectory models to identify distinct trajectories of BZD/non-BZD use over 12 months after initiation (i.e., trajectory period). We estimated risk of time to first occurrence of ADRD after trajectory period associated with these trajectories using inverse propensity score weighted Cox proportional hazards models. Among 131,574 beneficiaries (mean age=74 years, 68% female, 89% White), we identified 11 trajectories (4 BZD only [78% of cohort], 4 non-BZD only [18%], and 3 concurrent users [4%]). Compared to beneficiaries with low-dose discontinuing non-BZD only use, trajectories associated with an increased risk of ADRD included: stable moderate-dose BZD use with concurrent stable low-dose non-BZD use (HR=2.28, 95%CI=1.62-3.21) and high-dose declining BZD only use (HR=1.57, 95%CI=1.26-1.96). Results highlight a need to assess not only patterns of BZD and non-BZD use alone, but also concurrent use, when considering risk for ADRD.

